# Application of an Electrochemical Sensor Based on Nitrogen-Doped Biochar Loaded with Ruthenium Oxide for Heavy Metal Detection

**DOI:** 10.3390/bios15030160

**Published:** 2025-03-03

**Authors:** Le Li, Yonghong Zhao, Zhengjiu Wang, Jiale Tao, Manying Yang, Chen Li, Xiaoqian Zhang, Shiguo Sun, Na Zhao

**Affiliations:** 1 College of Chemical and Pharmaceutical Sciences, Northwest A&F University, Yangling 712100, China; teacher.lee@foxmail.com; 2 Key Laboratory of Xinjiang Phytomedicine Resource and Utilization, Ministry of Education, Department of Pharmacology, Shihezi University, Shihezi 832000, China; 13709930124@163.com (Y.Z.); wzj19990622@163.com (Z.W.); 18899122656@163.com (J.T.); 17690350659@163.com (M.Y.); 13333971289@163.com (C.L.); 20232015021@stu.shzu.edu.cn (X.Z.)

**Keywords:** cotton shell, electrochemical sensor, heavy metal, *Viola tianshanica* Maxim

## Abstract

Cotton is a widely cultivated cash crop and represents one of the most significant raw materials for textiles on a global scale. The rapid development of the cotton industry has resulted in the production of substantial amounts of cotton husks, which are frequently underutilized or discarded. This study utilizes agricultural waste, specifically cotton shells, as a precursor for biochar, which is subsequently carbonized and nitrogen-doped with ruthenium oxide to synthesize an innovative composite material known as RuO_2_-NC. An electrochemical sensor was developed using this composite material to detect heavy metals, particularly lead and copper ions. The results demonstrate that the electrochemical sensor can accurately quantify concentrations of lead and copper ions across a wide linear range, exhibiting exceptional sensitivity. Furthermore, the sensor was tested on samples from *Viola tianshanica* Maxim (Violaceae) collected from the Xinjiang Uygur Autonomous Region (XUAR) in China, showing commendable accuracy and sensitivity. This approach promotes eco-friendly recycling of agricultural waste while offering advantages such as straightforward operation and reduced costs, thereby presenting promising prospects for practical applications.

## 1. Introduction

In recent decades, significant advancements have been made in high-performance electrode material, with carbon materials garnering considerable attention due to their exceptional electrical conductivity, high specific surface area, and tunable porosity [1]. Three-dimensional porous carbon materials exhibit a large specific surface area, stable physical and chemical properties, and a three-dimensional interconnected porous structure, providing efficient channels for material transport, fluid flow, and gas diffusion [2]. However, the current precursors for these materials, such as activated carbon, carbon nanotubes, and graphene, are typically derived from non-renewable resources like coal or petrochemical products through complex and costly synthesis processes, which limit their widespread application.

The use of biomass materials as precursors for carbon materials offers several advantages, including environmental friendliness and sustainability. For instance, Ahmed El Nemr et al. investigated the utilization of a novel activated carbon, derived from date palm seed waste generated by the jam industry, for the removal of toxic chromium from aqueous solutions [3]. Rajan et al. developed activated carbon from date kernels, which boasts high carbon content, low ash content, and robust skeletal strength [4]. Similarly, Kurniawan et al. utilized waste tea leaves and coconut shells to produce activated carbon, demonstrating favorable adsorption performance, high mechanical strength, and cost-effectiveness [5]. Cotton, a vital cash crop, produces cotton shells as a byproduct during processing, which primarily consist of lignin (48.7%), cellulose (32.6%), hemicellulose (10.2%), and ash (5.8%). These shells are often discarded as waste, but recent research has focused on their adsorption capabilities, with limited exploration into their higher-value applications [6,7,8].

To enhance the value of cotton shell utilization, our research team developed a novel composite material RuO_2_-NC, by carbonizing cotton shells and incorporating nitrogen-doped ruthenium oxide. This material was then modified for use in an electrochemical sensor, enabling the quantitative detection of lead and copper ion concentrations in solutions. The sensor exhibits a broad linear concentration range and outstanding sensitivity. It has been successfully applied to the detection of lead and copper ions in samples, yielding promising results.

## 2. Materials and Methods

### 2.1. Characterization

Transmission electron microscopic (TEM) photographs were taken with an HT7700 transmission electron microscope (Hitachi, Tokyo, Japan) [9] via field emission scanning electron microscopy (SEM; Regulus8100, Hitachi, Tokyo, Japan) under magnifications of 10,000× and 50,000× at 5-kV accelerating voltage and 10.5 μA emission current. All the samples were fixed on double-side conductive tape and thus processed by gold sputtering in a vacuum (<1 Pa). X-ray diffractometry (XRD; D/max-2200PC, Rigaku, Tokyo, Japan) was employed to clarify the carbon crystal architectures and diffraction characteristic peaks of the samples under Cu Kα (λ = 0.154060 nm) radiation at 40 kV and 30 mA with a scan range and step size of 2θ = 10~80 and 0.020, respectively. The N_2_ adsorption/desorption isotherms were measured at 77 K to determine specific surface areas (SBETs), pore-size distributions (PSD), (BET; 3H-2000PM1, Beishide, Beijing, China). The SBET of each sample was analyzed from the isotherms (P/P0 = 0.0–1.0) through the Brunauer–Emmett–Teller (BET) method. The chemical speciation and mass compositions on the sample surfaces were analyzed by X-ray photoelectron spectroscopy (XPS; Thermo Scientific K-Alpha, Madison, WI, USA) under an ultra-high vacuum pressure of <1.0 × 10^−9^ Pa. High-resolution spectra were acquired using a monochromated Al Kα X-ray source (*hν* = 1486.6 eV) with a pass energy of 30 eV, a step size of 0.1 eV, averaged over 10 scans, and an X-ray power of 100 W (10 kV × 10 mA). Survey scans were recorded with a pass energy of 150 eV, a step size of 1.0 eV, and a dwell time of 50 ms [10,11].

### 2.2. The Preparation of Raw Materials

The cotton shell was crushed and sieved through a 100-mesh screen to obtain a fine powder. This fine powder was ultrasonically mixed with urea in water at a mass ratio of 1:6:10 (cotton shell fine powder/urea/water). The resulting suspension was dried to produce nitrogen-doped cotton shell [12]. Subsequently, the nitrogen-doped cotton shell was mixed with potassium bicarbonate in water, and the suspension was dried to obtain the modified cotton shell. The modified cotton shell was then mixed with ruthenium oxide at a mass ratio of 100:5 and placed in a tube furnace [13]. Under a high-purity nitrogen atmosphere, the mixture was heated to 800 °C for 2 h, yielding the nitrogen-doped biocarbon-loaded ruthenium oxide composite (RuO_2_-NC). Additionally, the fine cotton shell powder was subjected to pyrolysis in a tube furnace under the same conditions, followed by multiple washes and drying with water to obtain the bioC material. In the process of high temperature pyrolysis (800 °C, N_2_ atmosphere), the increased ruthenium oxide loading level (5 wt %) corroded the cotton husk due to the oxidation reaction catalyzed by RuO_2_, resulting in a significant reduction in the biochar content derived from it.

### 2.3. Preparation of Modified Electrodes

The bare glass carbon electrode was polished to a mirror-like finish using 0.3 μm and 0.05 μm alumina powder. Suspensions of bioC and RuO_2_-NC were prepared at a concentration of 5 mg/mL in ethanol. A 0.5% Nafion solution was added to the suspensions at a volume ratio of 0.04%. Ten microliters of each suspension was dropped onto the bare glass carbon electrode, and the solvent was allowed to dry; bioC/GCE-modified electrodes and RuO_2_-NC/GCE-modified electrodes were obtained. The RuO_2_-NC/GCE-modified electrode was characterized using scanning electron microscopy and transmission electron microscopy, and the electrochemical responses of the bare electrode, bioC/GCE-modified electrode, and RuO_2_-NC/GCE-modified electrode were compared. The electrochemical workstation used in this experiment was the CHI 660E Electrochemical workstation by Shanghai Chenhua Instrument Co., Ltd. (Shanghai, China).

### 2.4. Determination of Cu^2+^ and Pb^2+^ Solutions at Different Concentrations

Appropriate amounts of CuCl_2_ and Pb(NO_3_)_2_ were dissolved in an acetate buffer solution (0.1 M, PH = 4) to prepare solutions of varying heavy metal concentrations (Pb^2+^ and Cu^2+^). The electrochemical workstation was used for detection, with a saturated calomel electrode, platinum wire, and RuO_2_-NC/GCE-modified electrode serving as the reference, counter, and working electrodes, respectively. Metal ions were pre-deposited using the timed current method (*i-t*) at −1.1 V for 500 s, followed by detection via differential pulse voltammetry (DPV) in the potential range of −0.5 V to 0.8 V. A linear relationship graph was constructed based on the current intensity and the concentrations of Pb^2+^ and Cu^2+^.

### 2.5. Ion Selectivity Experiments

A solution of acetic acid buffer salt was spiked with 50 μM concentrations of lead, copper, sodium, potassium, nickel, iron, chromium, and aluminum ions. The pre-deposition was conducted using the chronocoulometric method, applying a deposition potential of −1.1 V for 500 s. Differential pulse voltammetry (DPV) was employed for the detection analysis.

### 2.6. Analysis of Samples

Dried *Viola tianshanica* Maxim rhizomes were crushed and sieved through an 80-mesh screen. One gram of the coarse powder was accurately weighed and placed in a Kjeldahl flask, to which 10 mL of a nitric acid–perchloric acid mixture (volume ratio 4:1) was added. The mixture was thoroughly mixed and left to soak overnight. It was then heated on an electric hot plate until it dissolved and turned brown–black. Additional nitric acid-perchloric acid mixture was added, and heating continued until the solution became clear. The temperature was increased, and heating continued until white smoke disappeared, leaving a slightly yellow digest solution. The container was washed with a 2% nitric acid solution, and the wash solution was combined with the digest solution in the flask, diluted to 250 mL, and thoroughly mixed to obtain the *Viola tianshanica* Maxim sample solution. Acetate buffer solutions containing different concentrations of heavy metals (Pb^2+^ and Cu^2+^) were added to the sample solution, and detection was performed using the electrochemical workstation.

## 3. Results

### 3.1. Structural Characterization of RuO_2_-NC-Modified Electrodes

#### 3.1.1. Physical Representation

**Figure 1:**
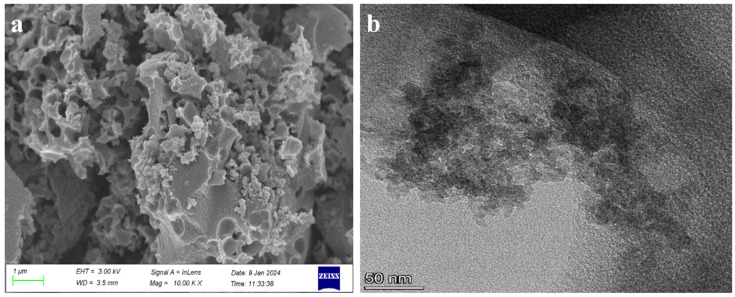
The image of the RuO_2_-NC composite material (**a**) Scanning electron microscopy (SEM) and (**b**) Transmission electron microscope (TEM).

The resultant RuO_2_-NC composites underwent physical characterization. Scanning electron microscopy (Figure 1a) reveals the porous structure of the cotton shell biochar and the adsorption of RuO_2_ on its surface. Owing to the elevated cohesive energy of Ru, agglomeration frequently occurs. The Figures demonstrate that RuO_2_ is uniformly distributed on the surface of the carbon material, forming Ru nanoclusters [14]. This significantly enhances the material’s surface area, providing additional binding sites for electrons and strongly supporting the optimization of material performance [15]. A transmission electron microscopic image (Figure 1b) shows that the RuO_2_-NC has a mesoporous structure [16].

**Figure 2:**
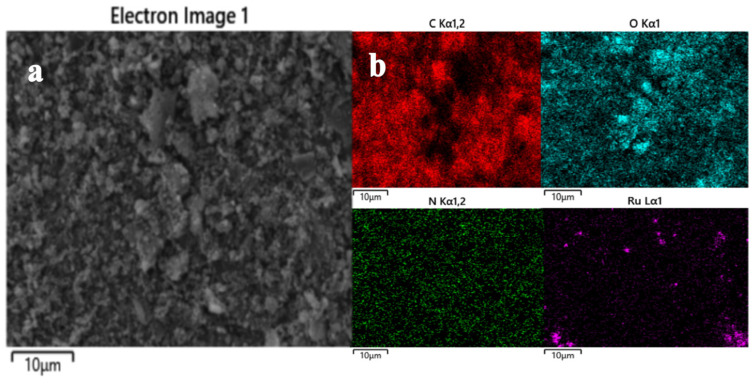
Elemental analysis diagram of RuO_2_-NC composite: (**a**) Backscatter micrograph of composite material; (**b**) Distribution of carbon, oxygen, nitrogen, and ruthenium elements.

For the RuO_2_-NC element mapping in the selected region, the corresponding energy-dispersive X-ray spectrum is presented in Figure 2, demonstrating the presence of carbon (C), oxygen (O), ruthenium (Ru), and nitrogen (N) elements in the material (Figure 2). This finding further confirms the uniform distribution of Ru nanoparticles on the surface of the modified cotton shell [17,18].

**Figure 3:**
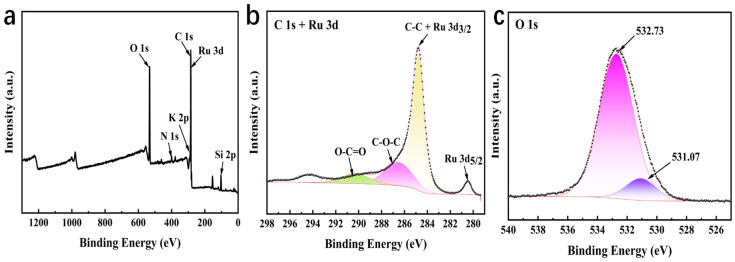
X-ray photoelectron spectroscopy (XPS) of RuO_2_-NC composites: (**a**) XPS spectrum; (**b**) C1s and Ru 3d XPS spectrum; (**c**) O1s XPS spectrum.

The chemical state and elemental composition of the RuO_2_-NC composite were characterized using X-ray photoelectron spectroscopy (XPS), as illustrated in Figure 3. Panel a represents the XPS spectrum, while panels b, and c, show the mapping images of individual elements [19]. Figure 3 reveals that RuO_2_-NC comprises C, N, O, and Ru elements. The C1s spectrum in Figure 3b exhibits peaks at 284.80, 286.53, and 290.24 eV, corresponding to C-C, C-O-C, and O-C=O bonds, respectively, which further confirms the successful synthesis of RuO_2_-NC composites [20]. Further analysis of the Ru3d core-level spectrum (Figure 3b) demonstrated characteristic spin–orbit splitting, with the Ru 3d_5/2_ and Ru 3d_3/2_ orbitals located at 280.18 eV and 284.80 eV, respectively, exhibiting a spin–orbit splitting of 4.62 eV. The observed binding energies align with the +4 oxidation state of Ru in the RuO_2_ lattice, while the slight positive shift (~0.3 eV) relative to pristine RuO_2_ suggests electronic interaction between the RuO_2_ nanoparticles and the nitrogen-doped carbon matrix. Notably, the overlap observed between the C-C binding energy (284.80 eV) and the Ru 3d_3/2_ orbital implies interfacial charge transfer and possible hybridization at the RuO_2_-NC heterojunction, likely arising from covalent bonding between Ru centers and oxygen-containing functional groups on the carbon scaffold during the composite formation process [21].

**Figure 4:**
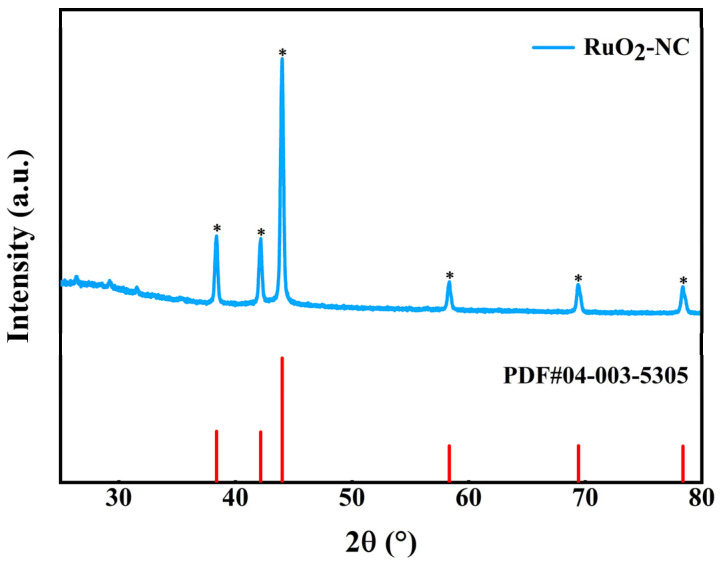
X-ray diffraction analysis of the RuO_2_-NC composite material. (”*”represents a peak corresponding to a standard PDF card).

The crystal structure of the RuO_2_-NC composite was further investigated using X-ray diffraction (XRD), as shown in Figure 4. The XRD pattern of RuO_2_-NC was compared with the standard PDF#04-003-5305 card, revealing strong and narrow diffraction peaks at 38.388°, 42.169°, 44.019°, 58.332°, and 69.424°, corresponding to the (100), (002), (101), (102), and (110) planes of hexagonal Ru crystals, respectively, confirming the successful synthesis of RuO_2_-NC composites [22].

**Figure 5:**
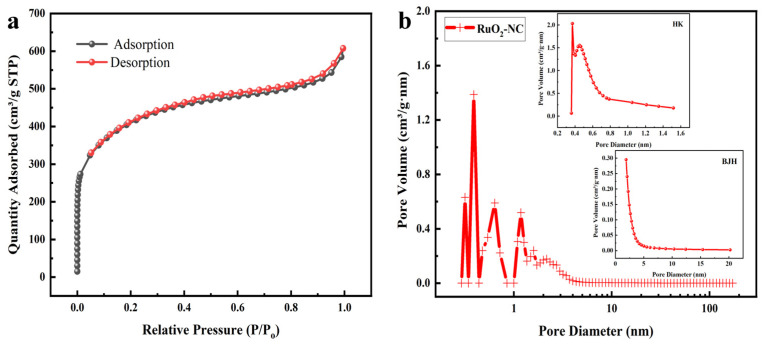
Nitrogen adsorption–desorption curve of RuO_2_-NC composite: (**a**) Nitrogen adsorption–desorption isotherm; (**b**) Aperture profile.

The nitrogen adsorption–desorption isotherm and the corresponding pore size distribution curve were analyzed using Brunauer–Emmett–Teller (BET) theory, as depicted in Figure 5. Figure 5a illustrates a Type IV isotherm with a hysteresis loop, characterized by a sharp adsorption step at low relative pressure (P/P0 < 0.01), a distinct capillary condensation step at intermediate relative pressure (0.5 < P/P0 < 0.8), and a tailing effect at high relative pressure (0.9 < P/P0 < 1.0) [23]. These features suggest the presence of a hierarchical pore structure comprising micropores, mesopores, and macropores. The pore size distribution, determined using density functional theory (DFT), Horvath–Kawazoe (HK), and Barrett–Joyner–Halenda (BJH) methods, is shown in Figure 5b, further supporting the presence of a hierarchical pore structure. The RuO_2_-NC composite exhibits a high BET specific surface area of 1471 m^2^/g and a total pore volume of 0.940 cm^3^/g, which facilitates the creation of a large electrochemically active region and enhances the exposure of electroactive sites within the pore channels [24,25].

#### 3.1.2. Electrochemical Characteristics

The electrochemical properties of biochar and RuO_2_-NC composites were evaluated using cyclic voltammetry (CV) and electrochemical impedance spectroscopy (EIS).

**Figure 6:**
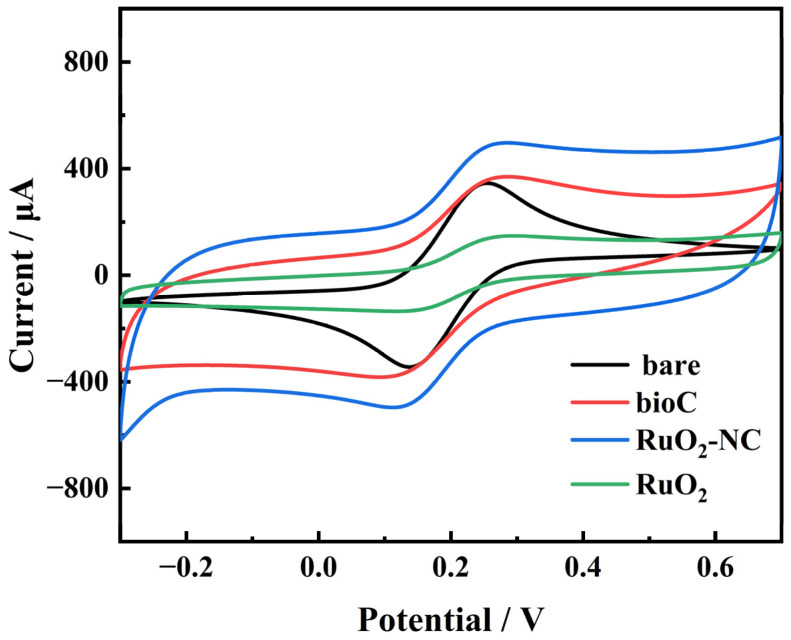
Cyclic voltammetry (CV) curves of the three prepared electrodes.

Figure 6 presents the CV curves of the bare glassy carbon electrode (GCE), bio/GCE, RuO_2_/GCE and RuO_2_-NC/GCE. Compared to the biochar material, The RuO_2_-NC composites exhibit distinct redox peaks originating from the [Fe(CN)_6_]^3−/4−^ couple. The peak current of the modified electrode is substantially higher compared to both the bare GCE and bioC/GCE. The results show that RuO_2_-NC/GCE has a larger electroactive surface area [26]. This enhanced performance can be attributed to the larger effective surface area provided by ruthenium oxide along with a high density of active sites, which collectively enhance the electrocatalytic activity of the electrode material [27,28].

**Figure 7:**
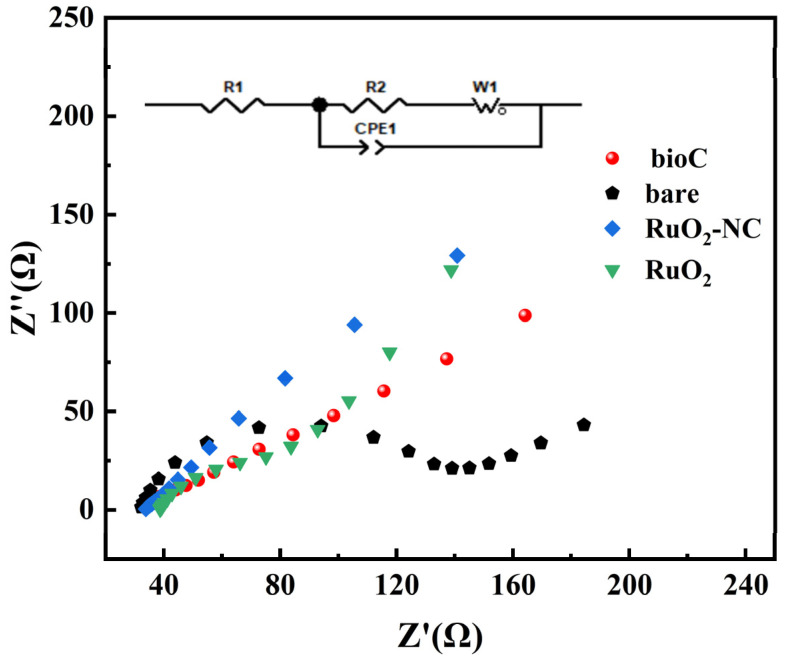
EIS plots of three prepared electrodes.

The EIS test results of the prepared electrode are shown in Figure 7. The impedance values are as follows: naked GCE impedance is 115 Ω, bioC/GCE impedance is 33 Ω, RuO_2_/GCE impedance is 38 Ω, and RuO_2_-NC/GCE impedance is 18 Ω. These findings show that with the addition of the modified material, the electrochemical impedance is gradually reduced, thus promoting the transfer of electrons. This result is consistent with the CV curve [29].

### 3.2. Linear Relationship Between Concentrations of Copper and Lead Ions

**Figure 8:**
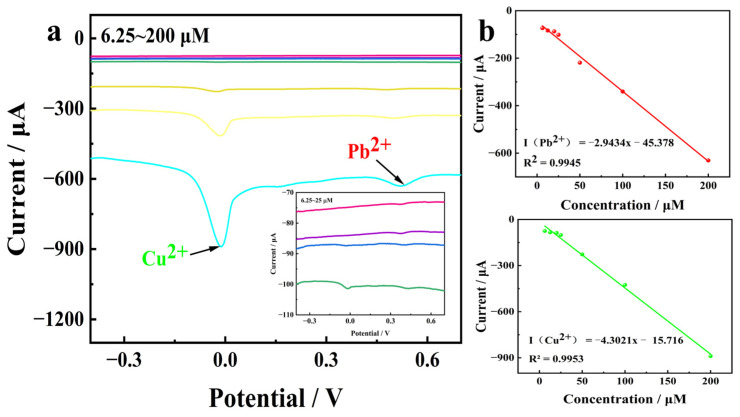
(**a**) Differential pulse voltammetry (DPV) simultaneous electroanalysis of Cd^2+^, Pb^2+^ at concentrations (6.25–200 μM) on the RuO_2_-NC. Every line in figure a from top to bottom in turn corresponding concentrations were 6.25 μM, 12.5 μM, 20 μM, 25 μM, 50 μM, 100 μM, 200 μM (**b**) Corresponding linear diagram (The green and red lines in the figure are linear relationships of Cu^2+^ and Pb^2+^, respectively).

Within the potential range of −0.5 V to 0.8 V, differential pulse voltammetry (DPV) was employed to record the corresponding voltammograms of Pb^2+^ and Cu^2+^ at various concentrations. The results are presented in Figure 8. It is evident that a strong linear relationship exists between the response current and ion concentration within the range of 6.25 to 200 μM. The detection limits for Cu^2+^ and Pb^2+^ were determined to be 0.039 μM and 0.051 μM, respectively [30].

### 3.3. Ion Selectivity Experiment Results

**Figure 9:**
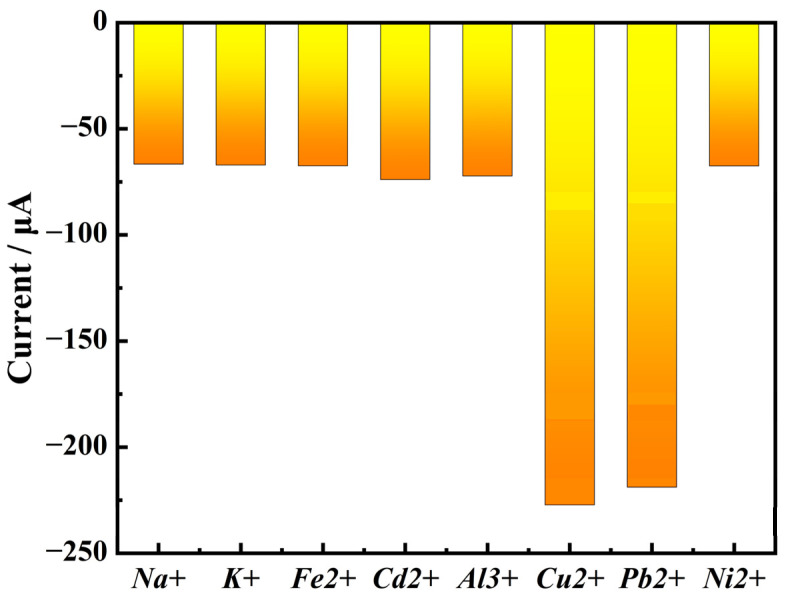
Electrochemical detector’s response to various metal ions.

As shown in Figure 9, the results show that the response current of RuO_2_-NC/GCE to Na^+^, K^+^, Fe^2+^, Cd^2+^, Al^3+^, and Ni^2+^ are less than half of that to Cu^2+^ and Pb^2+^ ions. Therefore, the electrochemical sensor modified with RuO_2_-NC composite material shows excellent selective detection ability of copper and lead ions [24,25].

### 3.4. Detection of Copper and Lead Ions in Samples by Electrochemical Sensor

Different concentrations of copper and lead ions were introduced into the sample solution and subsequently detected using the prepared electrochemical sensor. The recovery rates, as presented in Table 1, ranged from 95% to 105%. The average recovery of copper ion was 100.49 with an RSD(Relative Standard Deviation) of 1.63%, and the average recovery of lead ion was 101.27% with an RSD of 0.55%, These results indicate that the electrochemical sensor is suitable for the detection of copper and lead ions in samples.

## 4. Discussion

Global cotton production has exhibited a consistent upward trend in recent years. According to the latest data from the International Cotton Advisory Committee (ICAC), global cotton production for the 2024/2025 season is projected to reach 256.204 million tons, marking a 6.2% increase compared to the previous year. This growth can be attributed primarily to favorable climatic conditions in major cotton-producing regions and advancements in agricultural technology [31,32].

China stands as one of the world’s leading cotton producers, with Xinjiang serving as the country’s largest high-quality cotton cultivation base, accounting for over 80% of China’s total cotton planting area. The waste biomass, such as cotton husk derived from post-recycling cotton processing, possesses several advantages, including renewability, widespread availability, low cost, and environmental sustainability. Consequently, it has garnered increasing attention from researchers [33,34,35,36].

In this research, cotton husks were utilized as raw materials, carbonized, and subsequently doped with nitrogen and ruthenium oxide to develop a novel composite material. This material was employed to construct an electrochemical sensor for the detection of heavy metals, specifically lead and copper ions. The linear response range for Cu^2+^ and Pb^2+^ was determined to be 6.25–200 μM, with detection limits of 0.039 μM for Cu^2+^ and 0.051 μM for Pb^2+^. The sensor demonstrated strong anti-interference capabilities and was successfully applied to detect heavy metal ion residues in *Viola tianshanica* Maxim, indicating excellent application potential. This method leverages agricultural waste cotton husks as biochar material, simplifying the preparation process of the new composite material and promoting green recycling of agricultural waste, thereby representing beneficial exploration into the resource utilization of agricultural by-products.

## Figures and Tables

**Table 1:** *Viola tianshanica* Maxim test result.

Sample	Metal Ion	Background Quantity/μM	Added Quantity/μM	Measured Quantity/μM	Recovery %	Average Recovery %	RSD
*Viola tianshanica* Maxim	Cu^2+^	39.06	40	79.13	100.18	100.49	1.63%
			40	79.09	100.08		
			40	79.05	99.98		
			55	93.42	98.84		
			55	93.48	98.95		
			55	93.36	98.73		
			80	121.01	102.44		
			80	121.07	102.51		
			80	121.23	102.71		
	Pb^2+^	44.67	55	100.57	101.64	101.27	0.55%
			55	100.64	101.76		
			55	100.65	101.78		
			75	119.92	101.67		
			75	119.88	101.61		
			75	120.68	101.35		
			120	165.42	100.63		
			120	165.22	100.46		
			120	165.29	100.52		

## Data Availability

The original contributions presented in this study are included in the article. Further inquiries can be directed to the corresponding author.
